# What Is Meant by "Open Air"?

**Published:** 1898-07-30

**Authors:** 


### WHAT IS MEANT BY "OPEN AIR"?

What has been recently written about the advantages of the open-air treatment of consumption seems to render it desirable that no laxity should be allowed as to the precise meaning of the term, and that it should not be made to include methods to which it is not applicable in a strict sense. For example, a communication appeared a week or two ago in which a system of open-air treatment was described which involved the use of mechanical ventilation by steam-driven mechanism. No doubt this plan gave plenty of air, but the method could hardly be fairly called "open air" treatment. Again, in the current number of the *British Medical Journal* there is a very careful article on the subject by Dr. Philip, describing the open-air method adopted at the Victoria Hospital for Consumption, Edinburgh. In this he describes the rooms as having approximately 1,000 cubic feet o a per patient, as being heated by open fireplace, and as having the temperature regulated according to thermometric reading, a uniform temperature of 60 deg. F. being demanded, and it is stated that "most of the windows are provided with a closely-fitting board which is placed within the lower part of the framework, and on which the lower edge of the lower sash rests. This allows room in the stormiest weather for a free but gentle current of air to pass upwards between the sashes into the room." This, of course, is admirable, but a regulated temperature is hardly characteristic of open air, nor can we admit that the very useful plan of ventilation which is described has any part or lot in an open-air treatment. If we bear in mind that, ac-according to Pettenkofer, at the average velocity out of doors, 324,000 cubic feet of air flow over a person in the course of an hour, and compare that with the allowance of 3,000 cubic feet which is provided in the same time even in well-ventilated hospitals, and compare that again with what will filter in through a chink between an upper and a lower pane, we see how very different the conditions are. Certain effects have been stated to follow the treatment of consumption in the open air, but by this term is meant something more than open windows.

